# UHPLC-QQQ-MS/MS method for the simultaneous quantification of 18 amino acids in various meats

**DOI:** 10.3389/fnut.2024.1467149

**Published:** 2024-10-30

**Authors:** Mengxian Wang, Junxiu Guo, Huimin Lin, Dawei Zou, Jiaxuan Zhu, Zhenyuan Yang, Yufeng Huang, Fan He

**Affiliations:** ^1^Guangdong Provincial Hospital of Chinese Medicine, The Second Affiliated Hospital of Guangzhou University of Chinese Medicine, Guangdong, China; ^2^College of Chinese Medicine, Guangzhou University of Chinese Medicine, Guangdong, China

**Keywords:** amino acid, chemical analysis, pork, meat, UHPLC-QqQ-MS/MS, nutritional value

## Abstract

Amino acids are an essential source of human protein, and their content and composition are the main factors determining food protein utilization rate. Determining amino acids is essential in the component analysis of food. Therefore, a groundbreaking technique was developed utilizing ultra-high performance liquid chromatography interfaced with a triple quadrupole mass spectrometer (UHPLC-QQQ-MS/MS) for concurrently quantifying 18 amino acids across various types of meat. According to the test results, it can be known that the average content of glutamate (2.03 × 10^4^ ± 3.94 × 10^3^ μg/g in pig feet) was the highest in all meat samples, and the content of aspartate (0.0945 ± 0.0950 μg/g in pork) was the lowest, which was not detected in some samples such as beef and lean meat. Orthogonal partial least-squares discrimination analysis (OPLS-DA) showed: (1) 13 amino acids (arginine, valine, serine, alanine, lysine, glycine, asparagine, methionine, proline, threonine, glutamate, phenylalanine, and leucine, VIP > 1) were used as characteristic amino acids between pork and pig feet; (2) serine, threonine, alanine, histidine, asparagine, and arginine (VIP > 1) were used as signature amino acids in different components of pork (lean meat, fat, and pigskin); (3) asparagine, glutamate, histidine, tyrosine, and valine (VIP > 1) were considered as signature amino acids in different types of meats (pork, mutton, beef, and chicken). This study provides a new UHPLC-QQQ-MS/MS method for the determination of amino acid content in meat and also provides data support for the comprehensive evaluation of the nutritional value of foods containing amino acids.

## 1 Introduction

Amino acids (AAs) are the fundamental units of biological macromolecules such as peptides, proteins, enzymes, etc., and are a significant source of protein for human beings. Their content and composition are the major factors determining food protein utilization (1). The study of modern nutrition theory indicates that the deficiency or excessive amino acids influence the nutritive value of food (2). For the human body, 20 kinds of amino acids are required, of which nine are supplied by food, which the body cannot synthesize. These are called “essential amino acids” (EAAs), including tryptophane, lysine, threonine, methionine, leucine, isoleucine, valine, phenylalanine, and histidine. Among them, histidine is the essential amino acid for infants. Lack of EAAs can lead to abnormal physiological functions and diseases (3).

Similarly, insufficient or excessive intake of non-EAAs is harmful to the human body. Foods that maintain a similar proportion of amino acids to the human body’s requirements are considered highly nutritional (4). As an essential indicator of food quality, amino acids’ composition and content directly affect food protein quality. Therefore, analyzing the composition and content of amino acids in food is important.

Pork, pig feet, fat meat, lean meat, pig skin, beef, mutton, and chicken are some of the most readily available meats in the daily diet. Pork is the leading animal meat consumed in China (5). It is an excellent dietary source of high-quality protein and can provide rich EAAs for human beings. Amino acids are vital substances that reflect the nutritional value and taste characteristics of meat, and their content will change significantly with different parts, components, and types of meats. However, there is a lack of simultaneous comparative studies on amino acids in these meat samples. In addition, our research group is undertaking a mechanism study for a national-level project, speculating that amino acids in meat can reduce the toxic components of specific traditional Chinese medicines. So, we first conducted amino acid analysis on various meat samples.

In the analysis of food components, the determination of amino acid content is a vital part. Typically, amino acids are detected through an amino acid analyzer, high-performance liquid chromatography, and capillary electrophoresis. Zeng et al. developed a quantification method for 17 amino acids in tobacco leaves using amino acid analyzer and chemical spectrophotometry (6). Although the amino acid analyzer is convenient, it requires expensive equipment and has many limitations (7). Progress in mass spectrometry and separation techniques have propelled liquid chromatography–tandem mass spectrometry (LC–MS) to a vital status as an analytical method for amino acid identification. Zhao et al. successfully quantified 20 amino acids in cell culture media by the LC–MS method (8). Currently, the liquid chromatography tandem mass spectrometry (LC–MS) methods for amino acid analysis mainly focus on ultra-performance liquid chromatography–tandem mass spectrometry (UPLC–MS), high-performance liquid chromatography tandem mass spectrometry (HPLC–MS), and ultra-performance liquid chromatography quadrupole time-of-flight mass spectrometry (UPLC-Q-TOF-MS) (7, 9, 10).

In contrast, the ultra-performance liquid chromatography tandem triple quadrupole mass spectrometry (UPLC-QQQ-MS/MS) method has higher resolution, throughput, and quantitative accuracy than other LC–MS methods. Therefore, UPLC-QQQ-MS/MS was used to establish a quantitative method for determining 18 amino acids of different meat varieties in this study. Furthermore, the Amide column, a kind of column similar to a normal phase column, was used to simplify the pretreatment of the sample to be measured.

This investigation aims to elucidate the amino acid composition in various meats and introduce an innovative approach for concurrently quantifying 18 amino acids through UPLC-QQQ-MS/MS technology. This methodology contributes insights and techniques for the comprehensive assessment of the nutritional quality of meat derivatives, concurrently furnishing scientific references and empirical data to advance studies on amino acid-rich foods.

## 2 Materials and methods

### 2.1 Chemicals and reagents

Tryptophane, leucine, isoleucine, phenylalanine, aspartate, methionine, valine, tyrosine, proline, alanine, glycine, glutamate, threonine, serine, asparagine, arginine, histidine, lysine of purity no less than 98% were acquired from Shanghai Yuanye Company.

Formic acid, acetonitrile, and methanol were purchased from the Guangzhou chemical reagent factory, which were commonly used in the lab. Laboratory-grade ultra-pure water was supplied by a Milli-Q Synthesis purification system (Millipore, Billerica, MA, United States).

### 2.2 Apparatus and conditions

Agilent 6,460 triple quadrupole mass spectrometer (QQQ-MS, Agilent Technologies, Santa Clara, CA, United States) was used to quantitatively analyze 18 amino acids. Table 1 lists the optimal conditions for amino acid ionization, including parent and daughter ions, fragmentor voltage, and collision energy. The separation of compounds was executed using a Waters ACQUITY UPLC BEH Amide column (1.7 μm, 2.1 mm × 100 mm) at a constant temperature of 30°C. The mobile phase comprised 0.1% formic acid-water (A) and 100% acetonitrile (B), with a gradient elution program set as follows: 80 to 70% B from 0 to 7 min, after a 3-min post time. The flow rate maintained throughout the process was 300 μL/min. Eighteen amino acids were tested in positive ion mode using MRM and ESI sources. The remaining settings include a dry nitrogen gas flow of 11.0 L/min, a gas temperature of 300°C, a nebulizer pressure at 15 psig, and a capillary voltage for 4,000 V.

**Table 1:** Optimal conditions for amino acid ionization.

Amino acid	Parent ion (m/z)	Daughter ion (m/z)	Fragmentor voltage (V)	Collision energy (eV)
Tryptophane	205.1	146.1	60	14
Leucine	132.1	30.1	45	14
Isoleucine	132.1	86.1	65	10
Phenylalanine	166.09	120.1	70	14
Aspartate	134	30.1	134	14
Methionine	150.1	56.1	65	14
Valine	118.09	72.1	60	10
Tyrosine	182.08	91	70	30
Proline	116.1	70.1	70	14
Alanine	90.1	44.1	45	14
Glycine	76	30.1	40	14
Glutamate	148.06	84	85	14
Threonine	120.07	56.1	60	14
Serine	106.1	60.1	45	14
Asparagine	133.1	74	70	14
Arginine	175.1	70.1	105	18
Histidine	156.1	110	95	14
Lysine	147.1	84.1	65	14

### 2.3 Preparation of standard solutions

Tryptophane, leucine, isoleucine, phenylalanine, aspartate, methionine, valine, tyrosine, proline, alanine, glycine, glutamate, threonine, serine, asparagine, arginine, histidine, and lysine were weighed and prepared with methanol. Then precisely absorb the reserve solution of each single reference substance and mix it with methanol in an appropriate amount to obtain a mixed reference solution so that the concentration was as follows: 12.9 (Tryptophane), 44 (Leucine), 7.2 (Isoleucine), 25 (Phenylalanine), 1.74 (Aspartate), 0.636 (Methionine), 2.3 (Valine), 46 (Tyrosine), 15.4 (Proline), 18.6 (Alanine), 7.4 (Glycine), 25 (Glutamate), 7.2 (Threonine), 14 (Serine), 1.84 (Asparagine), 39 (Arginine), 3.3 (Histidine), 20 (Lysine) μg/mL, respectively.

### 2.4 Methodology for determination of amino acid content

#### 2.4.1 Linear relationship

Precise measurement of the mixed reference solution, dilution with chromatographic methanol step by step to obtain different gradient dilution solutions, placed in a refrigerator at 4°C, and a 0.22 μm filter membrane was employed to preprocess the sample before placement in the designated bottle. The mass spectrometry parameters were then utilized to assess the peak area of every constituent, and each concentration was injected three times in parallel. The peak area was plotted on the ordinate (y), while the known concentration of the amino acid standard was depicted on the abscissa (x). This facilitated the construction of the standard curve, upon which the regression equation was derived. According to the chromatographic conditions, the reference substance was diluted step by step and determined on the machine. The detection limit was established at the control concentration with a signal-to-noise ratio equivalent to 3 (S/N = 3). In contrast, the quantitative limit was set at the control concentration, exhibiting a signal-to-noise ratio of 10 (S/N = 10).

#### 2.4.2 Precision test

Consecutively measured the mixed reference solution six times according to 2.2 chromatographic conditions for determination, utilized the peak area of the sample to determine the individual amino acid content, and calculated the Relative Standard Deviation (RSD). The concentrations of amino acids in mixed reference solution were as follows: 3.23 (tryptophane), 11.0 (leucine), 1.80 (isoleucine), 0.581 (phenylalanine), 0.0544 (aspartate), 0.159 (methionine), 0.575 (valine), 11.5 (tyrosine), 3.85 (proline), 4.65 (alanine), 4.85 (glycine), 78.1 (glutamate), 1.80 (threonine), 3.50 (serine), 0.460 (asparagine), 9.75 (arginine), 0.825 (histidine), 5.00 (lysine) μg/mL, respectively.

#### 2.4.3 Repeatability test

Precisely weigh six copies of the same sample, adhere to the procedure outlined in section 2.3 to formulate the standard solution, determine the chromatographic conditions according to 2.2, and utilize the peak area to compute the individual amino acid concentration and the corresponding RSD in the sample.

#### 2.4.4 Stability test

The mixed reference solution was prepared according to the method under 2.3 and was placed at 4°C. Then, the solution was analyzed by UHPLC-QQQ-MS/MS at 0, 2, 4, 6, 8, and 10 h, respectively. The RSDs of the peak areas were calculated to verify the stability of various amino acids.

#### 2.4.5 Recovery test

The recovery test was repeatedly determined six times by spiking a certain amount (approximately 2.50 g) of a pork sample prepared by method 2.5.1 with a known amount of the mixed reference solution. The recovery rate was calculated according to the formula: recovery rate % = (total amount detected − original amount)/amount spiked × 100%. The recovery rates of the 18 amino acids should be within the range from 95 to 105%, and the RSD of the recovery rate of each compound should not be larger than 5%.

### 2.5 Determination of amino acid content

Precisely weighed six portions of pork, pig feet, fat meat, lean meat, pig skin, beef, mutton, and chicken, each 5.00 g. Minced and put them into a round-bottomed flask, respectively. Then, 50 mL of uncontaminated water was introduced into the flask, heated, and refluxed for 2 h after boiling. After cooling, the 200-mesh silk cloth was adopted for filtration, and the filtrate was finalized at 50 mL. Taking 400 μL of the extract and adding 1,200 μL of chromatographic methanol, vortexed, and the sample was subjected to centrifugation at 14,000 revolutions per minute for 10 min under a temperature of 4°C. Subsequently, the resulting supernatant was carefully filtered through an organic phase microporous filter membrane with a pore size of 0.22 μm in preparation for analysis. The injection volume was 2 μL.

### 2.6 Data analysis

A 6460 series triple quadrupole was operated with the assistance of Mass Hunter Workstation Software’s LC/MS Data Acquisition, specifically Version B04.01. For quantitative analysis in QQQ mode, Mass Hunter Workstation Software Quantitative Analysis Edition B 07.00/Build 7.0.57.0 was employed. The OPLS-DA analysis was executed utilizing SIMCA-P software (version 18.0, Umetrics, Umea, Sweden).

## 3 Results

### 3.1 Method validation

#### 3.1.1 Investigation of linear relationship

The standard curve uses each amino acid’s actual concentration (x) to make linear regression with the peak area (y), and Table 1 depicts the derived regression formula. It was observed that a commendable linear correlation existed for all 18 amino acids within their respective ranges. The details of the linear calibration curves, including the correlation coefficient (r), linear span, limit of detection (LOD), and limit of quantification (LOQ), are presented in Table 2.

**Table 2:** Calibration curves, linearities, LODs, and LOQs of the 18 reference compounds.

Amino acid	Linearity	*r*	Range (μg/mL)	LOD (ng/mL)	LOQ (ng/mL)
Tryptophane	y = 19334.21x-891.10	0.9998	0.0252–6.45	8.40	25.2
Leucine	y = 5099.67x + 1153.02	0.9999	0.172–44.0	7.16	21.5
Isoleucine	y = 143504.14x + 3576.30	0.9998	0.0228–7.20	0.469	1.41
Phenylalanine	y = 49362.57x-103.56	0.9992	0.0363–2.33	4.04	12.1
Aspartate	y = 7969.34x-23.96	0.9998	0.0136–0.218	4.53	13.6
Methionine	y = 3412.03x + 114.39	0.9999	0.0199–2.54	6.63	19.9
Valine	y = 86975.61x + 2341.35	0.9999	0.0359–2.30	0.598	1.80
Tyrosine	y = 296.92x + 315.78	0.9998	0.719–92.0	120	359.5
Proline	y = 108702.57x + 13241.88	0.9996	0.120–7.70	0.400	1.20
Alanine	y = 13546.92x + 315.78	0.9996	0.581–18.6	6.46	19.4
Glycine	y = 1315.40x + 123.11	0.9998	0.116–7.40	38.7	116
Glutamate	y = 3602.46x-3735.75	0.9994	9.76–625	2.08	6.25
Threonine	Y = 11321.28x + 277.01	0.9993	0.0141–7.20	4.67	14.1
Serine	y = 6918.78x-673.46	0.9999	0.438–14.0	7.30	21.9
Asparagine	y = 8750.90x-38.52	0.9999	0.0288–0.920	9.60	28.8
Arginine	y = 40463.73–13250.94	0.9999	0.609–19.5	101	304
Histidine	y = 32745.87x + 183.61	0.9998	0.0516–6.60	17.2	51.6
Lysine	y = 43408.99x-17645.45	0.9995	0.322–20.0	7.16	21.4

#### 3.1.2 Precision test

The RSD of the peak areas of the 18 amino acids was 0.365~4.75%, indicating that the instrument’s precision was good (Table 3).

**Table 3:** The precisions, repeatabilities, stabilities, and recoveries of the 18 reference compounds.

Analytes	Precision (RSD%, *n* = 6)	Repeatability (RSD%, *n* = 6)	Stability (RSD%, *n* = 6)	Recovery (*n* = 6)				
				Original (μg)	Spiked (μg)	Detected (μg)	Recovery (%)	RSD (%)
Tryptophane	3.58	4.86	2.88	6.08	6.04	12.2	102	2.81
Leucine	0.445	4.96	4.84	218	217	436	100	2.95
Isoleucine	0.365	4.96	4.36	20.3	20.2	40.8	101	2.08
Phenylalanine	1.67	4.97	4.67	8.32	8.20	16.6	101	2.92
Aspartate	3.75	4.21	3.93	0.288	0.297	0.576	97.1	4.32
Methionine	2.17	4.95	4.08	11.4	12.0	23.3	98.7	3.60
Valine	0.847	3.58	3.42	9.52	9.43	19.0	100	1.92
Tyrosine	1.10	4.00	4.67	292	290	581	99.4	3.27
Proline	1.96	4.30	3.14	39.8	39.0	79.0	101	3.14
Alanine	2.24	2.63	3.23	393	392	788	101	3.71
Glycine	2.24	2.96	3.28	66.0	65.6	131	99.2	3.29
Glutamate	4.64	3.29	4.71	3.24 × 10^3^	3.25 × 10^3^	6.48 × 10^3^	100	3.27
Threonine	2.03	3.53	4.18	75.6	75.2	151	100	2.02
Serine	1.41	4.73	3.60	62.0	61.6	125	102	4.10
Asparagine	3.61	4.37	4.78	13.9	14.0	27.6	97.7	1.02
Arginine	3.15	4.97	4.73	286	289	600	102	1.86
Histidine	4.75	3.77	4.30	250	249	500	101	2.12
Lysine	3.23	3.64	4.67	101	100	200	99.0	3.18

#### 3.1.3 Repeatability test

Six pork samples were accurately weighed, and adhering to the approach detailed in section 2.3, the test solution was prepared. Using the chromatographic settings outlined in section 2.2, the analysis was conducted on the instrument, resulting in a range of RSD values for the integrated peak areas of the 18 amino acids, varying from 2.63 to 4.97%, indicating that the sample reproducibility is good (Table 3).

#### 3.1.4 Stability test

The same standard solution under 2.3 was sucked up precisely and evaluated at hourly intervals from 0 to 10 h under the chromatographic parameters detailed in section 2.2. The RSDs for the peak area integrals of the 18 amino acids ranged from 2.88 to 4.84%. This demonstrates the acceptable stability of the test solution over 10 h, as the variations were within a limited range. The assessment confirmed the solution’s satisfactory stability during this time frame (Table 3).

#### 3.1.5 Recovery experiment

Recovery assessment involved the addition of pre-determined mixed standard quantities into samples (*n* = 6). The recovery rate (%) was calculated as follows: (sum of detected quantity after spiking − initial detected quantity)/spiked quantity × 100%. Table 3 illustrates that the recovery rates for the 18 amino acids ranged from 97.1 to 102%, accompanied by RSD values spanning 1.02–4.32%, which had high quantitative accuracy (Table 3).

### 3.2 Determination of amino acid content

**Figure 1:**
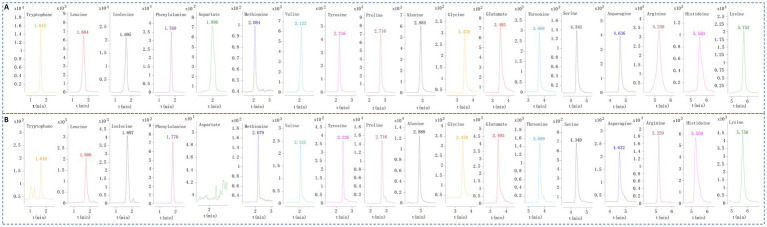
The representative EIC of the 18 amino acids of mixed reference solution (A) and pork test solution (B).

**Figure 2:**
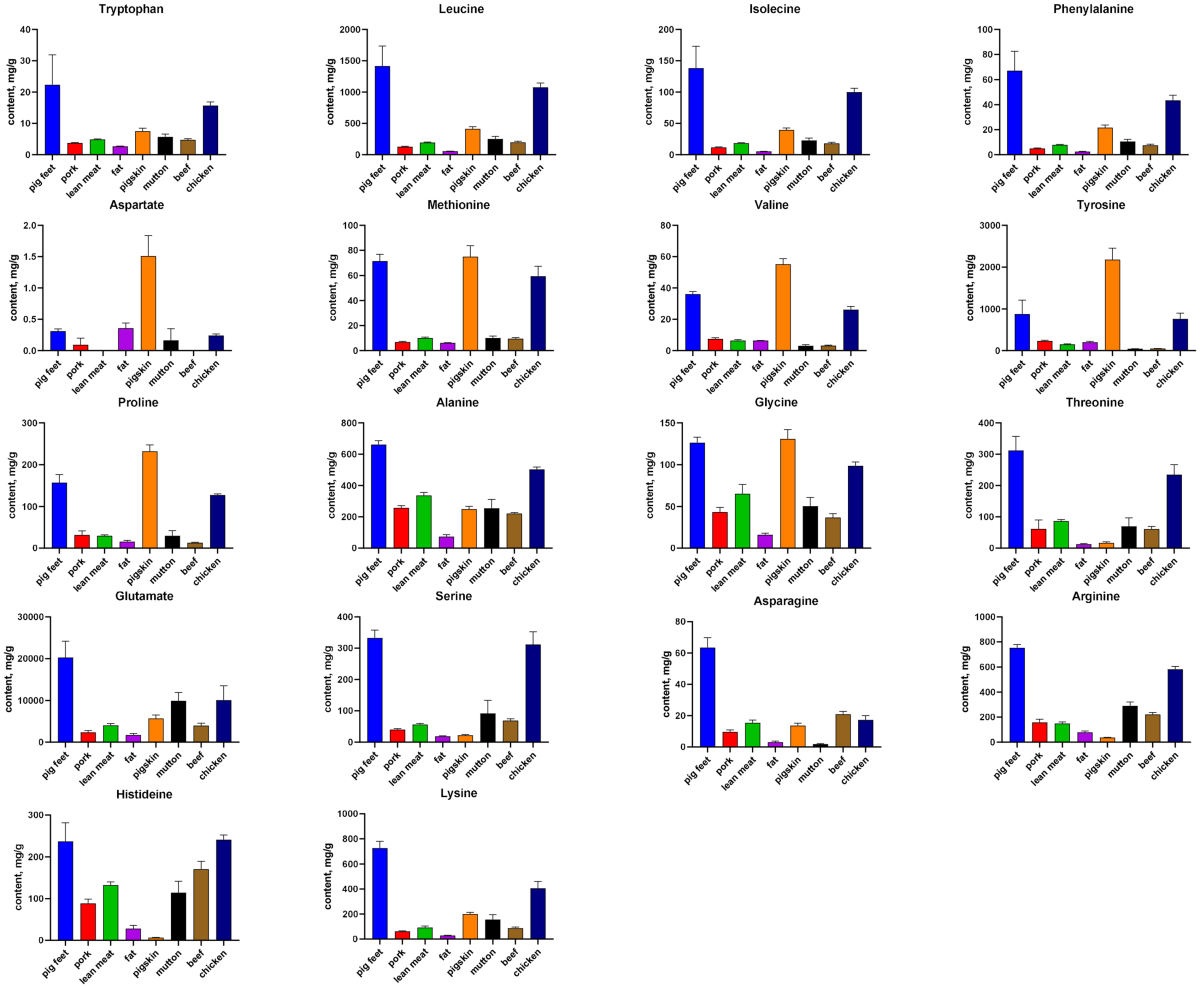
The distribution characteristics of the average content of 18 amino acids in different meats.

A chromatogram of standard amino acids and pork sample was shown in Figure 1. The data depicted in Figure 2 revealed that glutamate levels (2.03 × 10^4^ ± 3.94 × 10^3^ μg/g in pig feet) were the most abundant across all meat samples. The content of aspartate (0.0945 ± 0.0950 μg/g in pork) was the lowest, which was not detected in some beef and lean meat samples. Each amino acid content of pig feet was generally higher than that of pork’s group. In the fat, lean meat, and pigskin groups, the pigskin exhibited the highest concentration of total amino acids (9.39 × 10^3^ ± 9.14 × 10^2^ μg/g). The average content of various amino acids in chicken was higher than that in pork, beef, and mutton, except for beef’s asparagine (21.0 ± 1.71 μg/g).

### 3.3 Distribution of 18 amino acids in different samples

**Figure 3:**
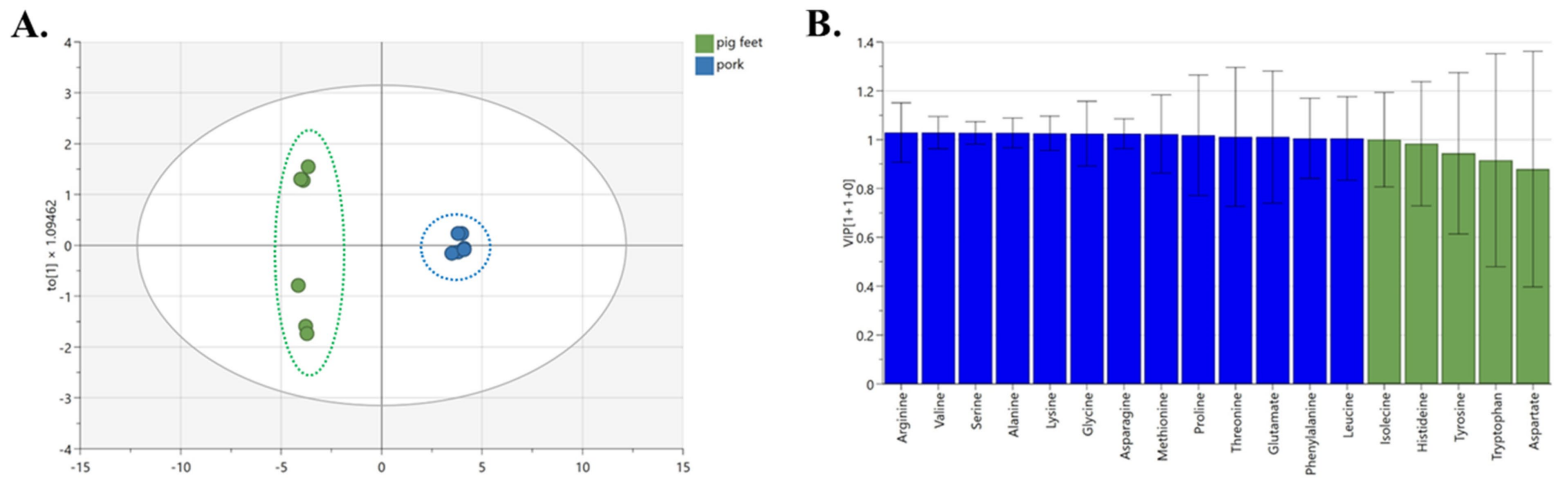
OPLS-DA analysis of different parts of pork samples. (A) OPLS-DA chart, (B) VIP chart.

The Orthogonal partial least-squares discrimination analysis (OPLS-DA) model shows that the clustering of pork and pig feet is significantly separated. The model exhibited an excellent fit with R^2^X, R^2^Y, and Q^2^Y values amounting to 0.972, 0.988, and 0.996, respectively, demonstrating strong predictability (Figure 3A). Thirteen amino acids (arginine, valine, serine, alanine, lysine, etc.) significantly influenced the grouping. They can be used as characteristic amino acids to distinguish pig feet from pork (Figure 3B, VIP >1).

**Figure 4:**
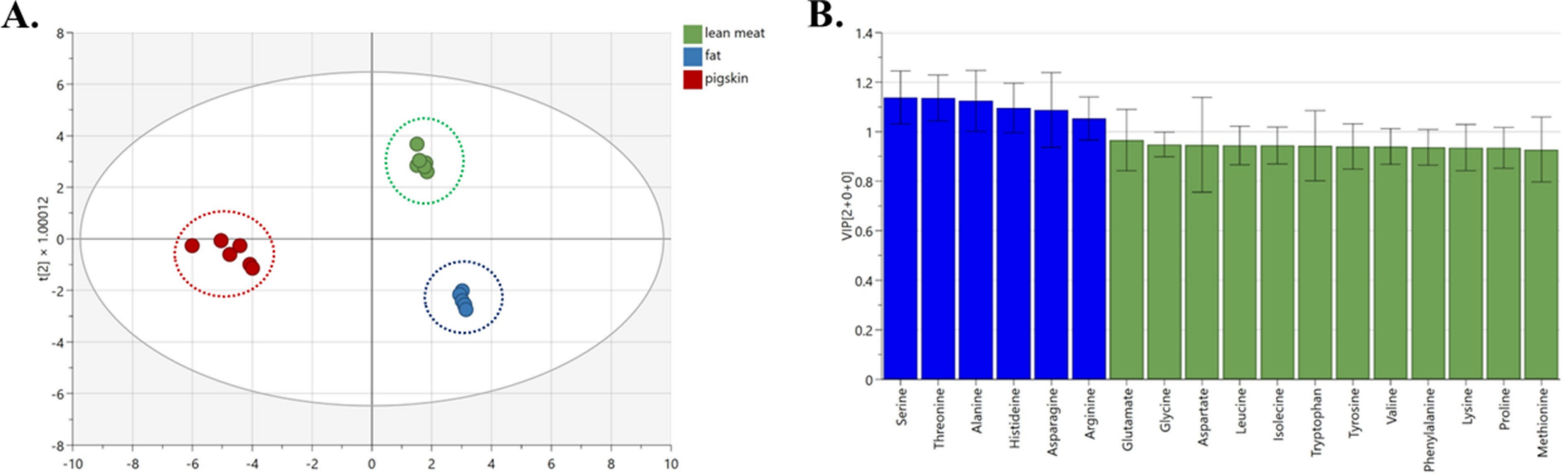
OPLS-DA analysis of different components of pork samples. (A) OPLS-DA chart, (B) VIP chart.

As depicted in Figure 4A, an absence of overlap was observed across the samples of fat, lean meat, and pig skin, indicating excellent discrimination. The model demonstrated excellent compatibility with an R^2^X value of 0.985, an R^2^Y of 0.983, and a Q^2^Y of 0.975, collectively suggesting a strong fit and satisfactory predictive capability. Six amino acids (serine, threonine, alanine, histidine, asparagine, and arginine) can be used as characteristic amino acids to distinguish the three different ingredients of pork (Figure 4B, VIP >1).

**Figure 5:**
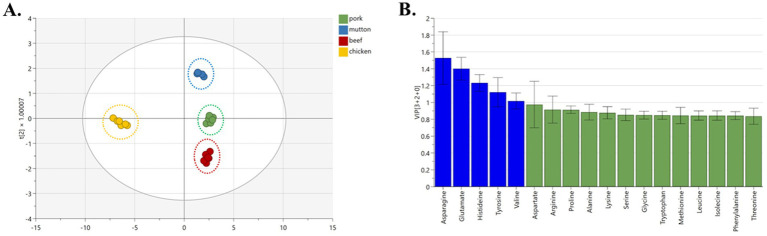
OPLS-DA analysis of different types of meat samples. (A) OPLS-DA chart, (B) VIP chart.

The OPLS-DA analysis reveals a clear distinction among samples derived from pork, beef, mutton, and chicken, forming distinct clusters visibly (Figure 5A). The model’s performance is attested by R^2^X, R^2^Y, and Q^2^Y coefficients of 0.992, 0.981, and 0.960, respectively, suggesting excellent model fitness and satisfactory predictive capability. Notably, the classification is significantly influenced by five amino acids, namely asparagine, glutamate, histidine, tyrosine, and valine (Figure 5B, VIP >1). The graphical representations of the OPLS-DA model consistently demonstrate optimal classification outcomes.

## 4 Discussion

A new UHPLC-QQQ-MS/MS approach was developed for the identification of 18 amino acids across various meat specimens in this investigation. The results showed this method was feasible, efficient, and sensitive. The analytical method we established has several advantages: (1) A newly styled chromatographic column (Waters ACQUITY UPLC BEH Amide column) was performed, which was more suitable for the separation of polar components (amino acids). When the common chromatographic column was used to determine amino acids by UHPLC–MS, the amino acid separation was ineffective (9). However, the chromatogram of this study shows good separation of 18 amino acids. (2) Sample pretreatment was simple in this study, reducing the loss of target components and better reflecting the actual situation of the sample. Previously, amino acid detection by LC–MS required complex sample pretreatment, such as urea derivatization (8) and hydrochloric acid hydrolysis (11). It enables the distinct segregation and quantitative determination of various amino acid categories within samples, thereby serving as an optimal selection for amino acid analysis.

Our research group consulted the literature in recent years and found that LC–MS mainly researched the hot topics on amino acid metabolism in pigs, beef, and chickens (12–14). The quantitative analysis of amino acids in meat was mainly to determine the content of total amino acids, free amino acids, or hydrolyzed amino acids in processed meat samples (15–17). The contents of specific amino acids in mutton were displayed in figures (18), which was unsuitable for verification with the 18 amino acids determined in this study. As we know, pig feet are rich in collagen, as are pig skin. In addition, the pig feet we weighed during sample processing did not contain bones, so, in this study, the total amino acid content of pig feet was higher than that of pig skin and lean meat for the above two reasons. The total amino acid content of lean meat was higher than that of pork and fat meat.

In comparing pig feet and pork, pig feet and pork samples could be well separated along PC1 (left and right) of the OPLS-DA analysis, which is related to the differences in the types and contents of amino acids. Among the pig feet samples, subgroups can be observed along PC2 (above and below), and the intuitive judgment is related to sample diameter, which needs further study. The glutamate, leucine, and lysine content in pig feet is higher than that of pork among the 13 characteristic amino acids screened out in this group. Glutamate is the basic component of human cells, which can promote cell metabolism and growth, especially for intestinal mucosal cells and immune cells (19). It can be converted into glucose in the body, providing the energy needed by cells, and is an important substance in energy metabolism (20). It also can activate the body’s immune system and enhance the function of white blood cells (21). Leucine helps prevent and relieve multiple liver diseases (22).

Meanwhile, lysine is an essential amino acid for enhancing the human immune system and is also very important for the human body. Lack of lysine will lead to significantly reduced human immunity (23). Therefore, it is suggested that pig feet be chosen preferentially because they are rich in glutamate, leucine, and lysine.

In comparing the different components of pork (fat meat, lean meat, and pig skin), the content of serine, threonine, and histidine in lean meat is significantly higher than that of the others, and these three are the different amino acids screened out in this group. Moreover, these three amino acids impact the human body, especially threonine and histidine, which are called EAAs that the human body cannot synthesize by itself and can only ingest from food. According to previous studies (24), animal proteins are regarded as the superior provider of indispensable amino acids due to their optimal absorption and utilization by the human physique. Notably, threonine plays a pivotal role in the metabolic equilibrium by converting specific amino acids (25); histidine is beneficial in regulating metabolism (26); serine can reduce the concentration of cholesterol in the blood and prevent high blood pressure (27). Therefore, lean meat rich in EAAs can be preferred as a nutritional supplement.

The histidine, tyrosine, and valine content were remarkably different among the five signature amino acids between pork and other meats (beef, mutton, chicken). These three are also essential nutrients in the human body. Tyrosine is an important component of thyroxine, which has an important relationship with the transmission of neurons and helps to improve the body’s immunity (28). Valine can reduce capillary permeability, reduce brain tissue edema, and improve microcirculation (29). It also has a specific role in promoting the rehabilitation of the nervous system (30). According to the comprehensive evaluation of nutritional value, patients with a corresponding amino acid deficiency can choose to eat more chicken first.

## 5 Conclusion

This study established a novel method for simultaneously determining 18 amino acids in different meats after cooking for 2 h based on UHPLC–QQQ–MS/MS, and its rapid, sensitive, and accurate characteristics were verified. Then, combined with the OPLS-DA model, the differential amino acids between meat products were screened. The approach was effectively utilized for a comparative assessment of amino acid concentration variations. Findings indicated substantial differences in amino acid levels across various meat sections, compositions, and types, offering insights and strategies for comprehensively appraising meat’s nutritional worth. In addition, this method provides great application value in protein chemistry, food science, and clinical medicine.

## Data Availability

The original contributions presented in the study are included in the article/supplementary material, further inquiries can be directed to the corresponding authors.
